# Comparative Evaluation of Three Primary Antibody Clones for p16 Immunohistochemistry in Gynecologic Tumors

**DOI:** 10.3390/antib14030077

**Published:** 2025-09-05

**Authors:** Hiroshi Yoshida, Ayumi Sugitani, Mayumi Kobayashi-Kato, Masaya Uno, Mitsuya Ishikawa

**Affiliations:** 1 Department of Diagnostic Pathology, National Cancer Center Hospital, 5-1-1 Tsukiji, Chuo-ku, Tokyo 104-0045, Japan; 2 Department of Gynecology, National Cancer Center Hospital, 5-1-1 Tsukiji, Chuo-ku, Tokyo 104-0045, Japan

**Keywords:** p16, immunohistochemistry, antibody clone, gynecologic cancer, interobserver reproducibility

## Abstract

Background: p16 immunohistochemistry (IHC) serves as a surrogate marker for high-risk human papillomavirus (hrHPV) and is widely used in gynecologic pathology. However, few studies have directly compared the staining performance and reproducibility of different p16 antibody clones in this context. Methods: We retrospectively evaluated 176 gynecologic tumor specimens including 42 whole slide sections and 134 tissue microarray cores from the cervix, endometrium, vulva, and ovary using three fully automated p16 IHC assays: E6H4 (Ventana/Roche), JC8 (Agilent/Dako), and 6H12 (Leica). Two pathologists independently reviewed each case, and concordance and interobserver agreement were analyzed. Sensitivity, specificity, and Cohen’s κ statistics were calculated, with E6H4 serving as the reference. Results: All three antibody clones demonstrated excellent staining performance with preserved tissue morphology and minimal background artifacts. Concordance for p16 positivity/negativity was 100% across all clone pairings (95% CI: 97.9–100%). Interobserver reproducibility was also perfect, with a κ coefficient of 1.00 (95% CI: 0.94–1.00). Minor non-block staining patterns did not impair interpretability. Conclusions: Our findings indicate that E6H4, JC8, and 6H12 clones yield comparable staining results when used in conjunction with standardized automated protocols. These results support the practical interchangeability of these clones in clinical and research settings, particularly when cost, availability, or risk management require substitution. Laboratories should continue to perform internal validation and utilize external quality assurance programs when implementing p16 IHC.

## 1. Introduction

Immunohistochemical staining (IHC) for p16INK4a (CDKN2A) is the most widely employed surrogate marker for reflecting genomic integration of high-risk human papillomavirus (hrHPV) in formalin-fixed, paraffin-embedded (FFPE) tissues [1]. In contemporary pathology practice, it has become an essential diagnostic tool, particularly in tumors of the lower anogenital tract [2] and head and neck regions. The p16 protein functions by binding to cyclin-dependent kinases 4 and 6 (CDK4/6), thereby maintaining the retinoblastoma protein (pRb) in a hypophosphorylated state [3,4]. This hypophosphorylated pRb sequesters E2F transcription factors, preventing cell cycle progression. In HPV-associated neoplasms, viral integration into the host genome leads to the expression of the viral oncoproteins E6 and E7 [5]. E6 promotes the degradation of p53, thereby inhibiting apoptosis [5]. The E7 oncoprotein functionally inactivates pRb, preventing it from binding to E2F transcription factors. As a result, a positive feedback loop leads to the accumulation of p16 protein in both the nucleus and cytoplasm, which can be visualized by immunohistochemistry (IHC) [1,6]. Transcriptionally active HPV is known to induce markedly high levels of p16 expression [7], whereas the E7 protein encoded by low-risk HPV types possesses distinct functional characteristics [8]. Consequently, p16 accumulation is generally absent in cells infected with low-risk HPV [9]. Thus, p16 overexpression detectable by IHC is widely used as a surrogate marker for the presence of hrHPV in tumors, particularly cervical neoplasms [1,2]. In cervical and vulvar carcinomas, HPV status has been reported as a prognostic factor [10], and p16 IHC may serve as an important biomarker for risk stratification of recurrence and for informing therapeutic decision-making in the future.

The clinical utility of p16 IHC extends beyond its role as a surrogate marker for hr HPV infection. Strong and diffuse p16 expression is commonly observed in endometrial serous carcinoma as well as in high-grade serous carcinomas arising from the fallopian tube and ovary [11]. Similar staining patterns have also been reported in uterine carcinosarcomas and undifferentiated carcinomas [12,13]. In such cases, p16 IHC may provide supportive evidence for histotype identification. When a diagnosis of high-grade carcinoma is rendered, these findings may influence surgical decision-making and the administration of adjuvant therapy according to the predicted risk of recurrence. Therefore, p16 can be considered a clinically significant biomarker in this context. Interestingly, the mechanism underlying p16 overexpression in these tumors appears to be unrelated to HPV infection. Disruption of the Rb pathway, such as point mutations or deletions of the RB1 gene or genetic alterations like CCNE1 amplification, is thought to be responsible for p16 overexpression in these malignancies [11,14].

Thus, p16 IHC is widely recognized as a clinically significant diagnostic marker in gynecologic pathology. Multiple primary antibody clones targeting p16 are commercially available [1], and several reports have indicated that staining characteristics may vary depending on the specific clone used [15]. However, despite the increasing availability of fully automated IHC systems, comparative data on staining performance using different commercially available p16 antibodies remain limited, particularly in gynecologic tumors. Such data are essential for laboratory risk management—for example, in the event of performance issues with a specific clone—as well as for assessing the comparability of research findings that utilize different antibodies. Moreover, these comparisons are critical for evaluating the cost-effectiveness of p16 IHC protocols in clinical practice.

The present study aims to compare the staining characteristics of three widely used [16] and fully automated p16 antibody clones—E6H4 (Ventana/Roche), JC8 (Agilent/Dako), and 6H12 (Leica biosystems)—using gynecologic tumor specimens. Our goal is to provide foundational data to support laboratory decision-making regarding clone substitution, assay standardization, and performance optimization in the context of gynecologic pathology.

## 2. Materials and Methods

### 2.1. Patients and Study Design

We conducted this retrospective study in accordance with the Declaration of Helsinki. The study was approved by the Institutional Review Board of the National Cancer Center, Japan (#2020-111, 2010-077). From January 2023 to May 2025, we included 42 cases of gynecological tumors, including cervical cancer, vulvar cancer, endometrial cancer, and ovarian cancer, who underwent surgery at the National Cancer Center Hospital, Tokyo, Japan. Due to the retrospective nature of this study, obtaining individual informed consent from participants for study participation was waived by the Japanese ethics guidelines [17]. Informed consent for using samples in research was obtained through general informed consent.

A total of 42 cases were selected from routine diagnostic practice, in which immunohistochemical staining had been performed using the E6H4 clone (Ventana/Roche) according to the protocol described below. E6H4 clone is contained in CINtec p16 IHC assay which has been approved (FDA Class II IVD device) as a surrogate marker for HPV in FFPE cervical biopsy samples on the BenchMark staining platform [18]. The selected cases included 21 positive and 21 negative cases, as determined independently by at least two board-certified pathologists. Tumors were chosen to represent a variety of gynecologic sites and histologic subtypes (Table 1). In accordance with the College of American Pathologists (CAP) guidelines for validation of IHC [19], a minimum of 10 positive and 10 negative cases are recommended for non-predictive markers, and at least 20 positive and 20 negative cases for predictive markers. Based on these recommendations, the above sample size was determined.

In addition, tissue microarrays (TMAs) comprising 44 cases of endometrial carcinoma and 90 cases of ovarian carcinoma were used for further validation (Table 2). TMAs were constructed by selecting representative 3 mm cores for endometrial carcinomas and 1.5 mm cores for ovarian carcinomas from formalin-fixed, paraffin-embedded tumor blocks. Unlike the 42 whole slide cases described above, these TMAs had not been previously stained for p16; therefore, all three p16 antibody clones were applied.

All tissue specimens had been fixed in 10% neutral buffered formalin for 12 to 48 h, and none had undergone decalcification. From FFPE tissue blocks, 4–5 µm thick unstained sections were serially cut, and all IHC staining procedures were completed within three weeks of sectioning.

### 2.2. IHC Procedure

The details of the p16 immunohistochemical staining protocols used in this study are summarized in Table 3. Three primary antibody clones—E6H4 (Ventana/Roche Diagnostics), JC8 (Agilent/Dako), and 6H12 (Leica)—were employed according to the respective manufacturers’ instructions. We selected these 3 clones because they have been widely used. In NordiQC’s p16 run 72 (2024), a total of 431 laboratories participated. Among the 357 laboratories using ready-to-use (RTU) antibodies, the top three antibodies employed were E6H4 (Ventana/Roche) in 77% (274/357), JC8 (Agilent/Dako) in 7.6% (27/357), and 6H12 (Leica) in 7.6% (27/357) of the laboratories [16]. Each antibody was applied using the vendor-recommended automated staining platforms and protocols: BenchMark ULTRA (Ventana/Roche Diagnostics) for E6H4, Dako Omnis (Agilent/Dako) for JC8, and Leica BOND III (Leica biosystems) for 6H12.

## 3. Interpretation

All p16 IHC stains were evaluated alongside their corresponding hematoxylin and eosin (H&E)-stained sections. Initially, positive and negative control slides were reviewed to confirm the integrity of the staining procedure and to assess for any non-specific staining. For each case, the stained section was first examined to ensure that tissue morphology was adequately preserved and that no non-specific staining was present. Subsequently, the staining pattern in tumor cells was evaluated.

Based on the previous studies, IHC was interpreted as positive when moderate to strong nuclear and cytoplasmic staining was observed in ≥70% of the tumor cells throughout the section [1]. Accordingly, each case was classified into one of the following categories: Positive (block-type positivity), Negative (including mosaic-type staining or absence of staining), or Uninterpretable.

For the assessment of interobserver reproducibility, two pathologists independently evaluated each case for the presence or absence of p16 positivity using each antibody clone. A total of 60 slides were evaluated, including 20 cases stained with each of the three antibody clones (10 positive and 10 negative per clone). The evaluators were blinded to all clinical information and histopathologic diagnoses.

### Statistical Analysis

Baseline characteristics were presented as frequencies and proportions for categorical variables. Interobserver agreement was calculated using Cohen’s κ (kappa) statistics. In cases of discordant interpretation, both observers jointly reviewed the slides to determine the source of disagreement. The sensitivity and specificity of each antibody clone were calculated using standard 2 × 2 contingency tables, with E6H4 considered as the provisional reference standard. Statistical differences were considered significant when the 2-sided *p*-value was less than 0.05. All statistical analyses and graphic presentations were performed with SPSS (version 13.0J; SPSS Inc., Chicago, IL, USA).

## 4. Results

All 42 cases assessed by whole slide sections and 134 cases evaluated by TMAs were included in the analysis. The organ of origin, pathological diagnosis, specimen type (surgical resection vs. biopsy), and staining pattern using the E6H4 clone are summarized in Table 1. A total of 71.4% (30/42) of the specimens were derived from cervical tumors, and 61.9% (26/42) were surgical resection specimens. The cohort included an equal number of p16-positive and p16-negative cases (21 each), as determined by staining with the E6H4 clone. The most common diagnosis was HPV-associated squamous cell carcinoma, accounting for 12 of the 42 cases (28.6%).

**Figure 1:**
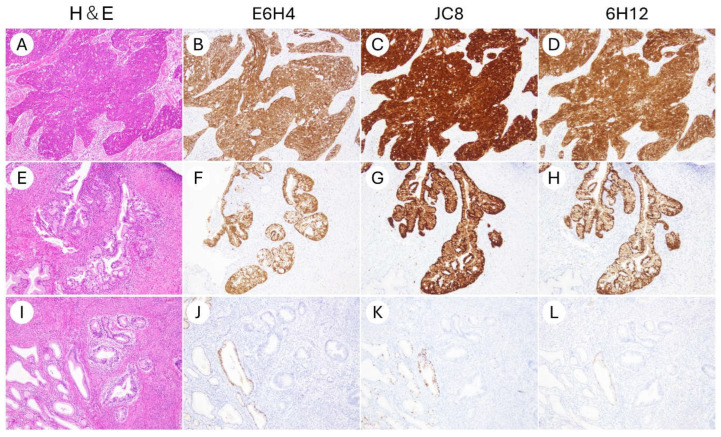
Representative micrographs of p16 immunohistochemical staining using three different antibody clones in uterine cervical cancers (×200). (**A**–**D**) HPV-associated squamous cell carcinoma of the uterine cervix. (**E**–**H**) HPV-associated endocervical adenocarcinoma. (**I**–**L**) HPV-independent endocervical adenocarcinoma. Clones E6H4, JC8, and 6H12 exhibited highly comparable staining patterns. Block-type nuclear and cytoplasmic positivity was consistently observed in HPV-associated tumors (**B**–**D**,**F**–**H**), whereas HPV-independent tumors showed negative staining (**J**–**L**).

**Figure 2:**
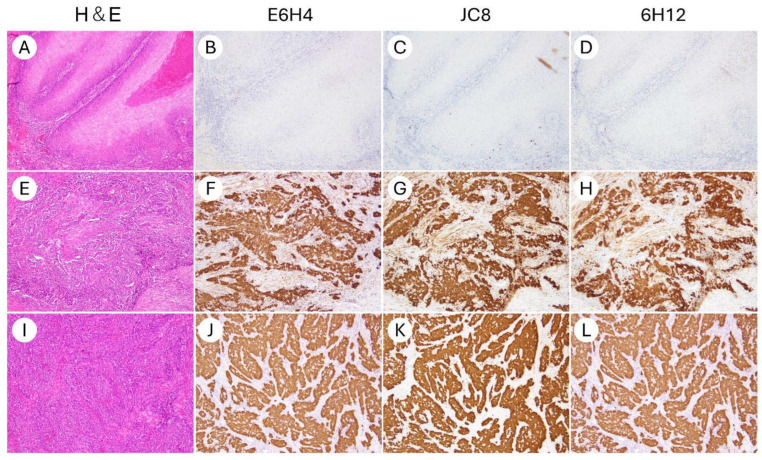
Representative micrographs of p16 immunohistochemical staining using three different antibody clones in non-cervical gynecologic cancers (×200). (**A**–**D**) HPV-independent vulvar squamous cell carcinoma. (**E**–**H**) Ovarian high-grade serous carcinoma. (**I**–**L**) Uterine serous carcinoma. Clones E6H4, JC8, and 6H12 demonstrated consistent staining patterns across all cases. HPV-independent vulvar squamous cell carcinoma was negative for p16 expression (**A**–**D**), whereas block-type nuclear and cytoplasmic positivity was observed in the serous carcinomas of the ovary and endometrium (**E**–**L**).

In all three clones, IHC for p16 was technically successful, with no issues observed in the staining process or the performance of the positive and negative controls. Tissue morphology was well-preserved in all cases, allowing for a reliable interpretation. No slides were excluded due to non-specific staining or artifact-related interpretability issues. Representative staining patterns for each clone are shown in Figure 1 and Figure 2.

Next, the staining characteristics of each clone were compared (Table 4). The three clones—E6H4, JC8, and 6H12—demonstrated comparable staining performance. The concordance rate for p16 positivity/negativity was 100% (95% confidence interval: 91.6–100.0%) for all pairwise comparisons: E6H4 vs. JC8, E6H4 vs. 6H12, and JC8 vs. 6H12.

Using E6H4 as the reference standard, both JC8 and 6H12 clones demonstrated a sensitivity of 100% (21/21; 95% confidence interval: 84.5–100.0%) and a specificity of 100% (21/21; 95% confidence interval: 84.5–100.0%).

**Figure 3:**
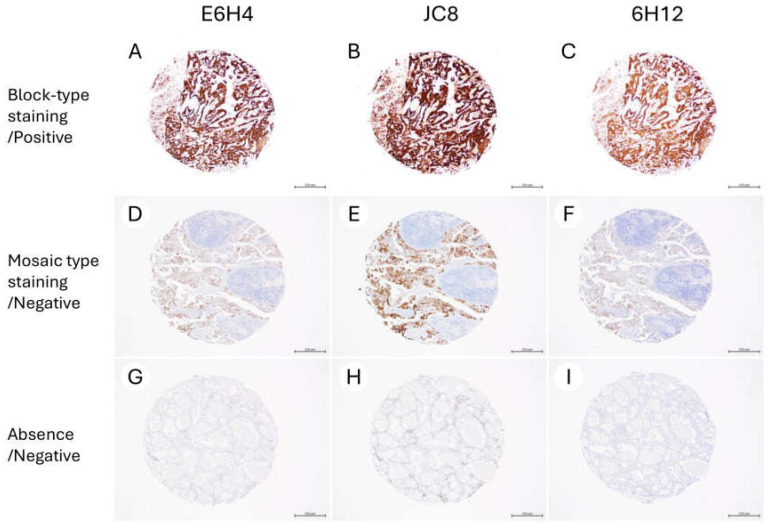
Representative micrographs of p16 immunohistochemical staining using three different antibody clones in tissue microarrays (×40). (**A**–**I**) (**A**–**C**) High-grade serous ovarian carcinoma shows block-type p16 positivity. (**D**–**F**) Endometrioid carcinoma shows mosaic-type staining pattern. (**G**–**I**) Clear cell carcinoma presents absence of p16 staining. Clones E6H4, JC8, and 6H12 demonstrated consistent staining patterns across all cases. A scale bar indicates 500 µm.

For further validation, TMAs including 44 endometrial cancers and 90 ovarian cancers were stained using all the 3 clones (Table 5). As observed in the whole slide sections, staining results of 3 different clones were concordant (Table 5). The representative findings are shown in Figure 3.

To assess interobserver reproducibility, 60 specimens were independently evaluated by two board-certified pathologists (Table 6).

The results showed complete concordance in the classification of p16 immunoreactivity (positive vs. negative) across all 60 slides. The overall concordance rate was 100%, with a Cohen’s κ coefficient of 1.00 (95% confidence interval: 0.94–1.00).

**Figure 4:**
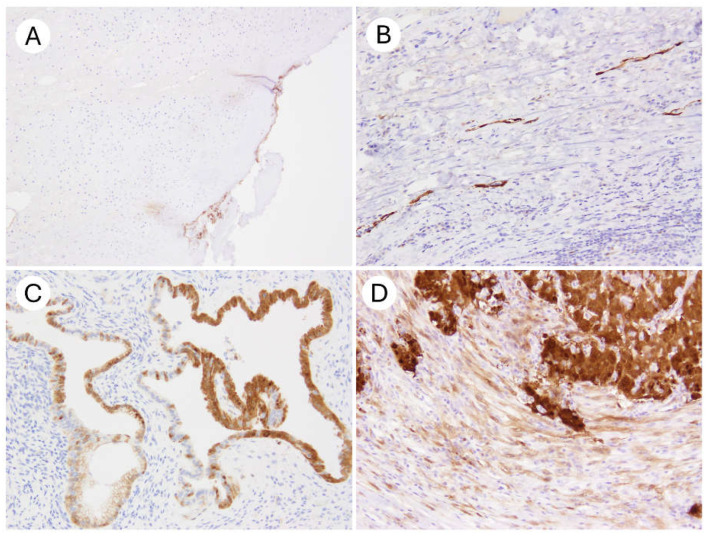
Examples of non-specific p16 immunohistochemical staining. (**A**) Edge artifact along the tissue margin (×100). (**B**) Aberrant p16 positivity in lymphatic endothelial cells (×200). (**C**) Mosaic p16 staining in tubal metaplasia of endocervical glands (×200). (**D**) Non-specific stromal staining observed at the invasive front (×200).

Concerning non-specific staining, minor degrees of edge artifacts, positive staining in tubal-type metaplastic epithelium, and occasional stromal cell positivity were observed across all antibody clones to varying extents (Figure 4).

However, when the established cytomorphologic criteria and diagnostic thresholds for p16 positivity and negativity were applied, these background findings did not interfere with the interpretability of the IHC results.

## 5. Discussion

In the present study, we compared the staining performance of three fully automated p16 IHC assays—E6H4, JC8, and 6H12—across 176 gynecologic tumor specimens, including cases from the cervix, endometrium, ovary, and vulva. All three antibody clones demonstrated complete concordance in classifying p16 positivity and negativity, with excellent interobserver reproducibility.

It has been reported that the performance of p16 IHC may vary depending on the antibody clone used, highlighting the importance of comparative evaluation between different clones. Shelton et al. investigated 199 cases of oropharyngeal squamous cell carcinoma (OPSCC) and compared the performance of three p16 clones: E6H4 (Roche CINtec), JC8 (Santa Cruz), and G175-405 (BD) [20]. The positive predictive values (PPVs) for high-risk HPV positivity among p16-positive cases were 98% for E6H4, 100% for JC8, and 99% for G175-405, using a 75% cutoff. However, notable differences were observed in negative predictive values (NPVs): 86% for E6H4, 69% for JC8, and 56% for G175-405 [20]. E6H4 also demonstrated the highest staining intensity (3+ staining in 85% of cases, compared to 72% for JC8 and 67% for G175-405). Furthermore, interobserver agreement and correlation with patient survival were highest for E6H4. Based on these findings, Shelton et al. concluded that the E6H4 clone exhibited superior performance, with less background staining, better quantifiability, stronger prognostic correlation, and greater reliability compared to the other clones [20].

In contrast, our study demonstrated a high degree of concordance in staining results between E6H4 and JC8. Moreover, unlike Shelton et al., we found that JC8 produced stronger staining than E6H4 in our specimens (Figure 1). One possible explanation for this discrepancy may lie in the differences in antibody source and staining protocol. In Shelton’s study, the JC8 clone was obtained from Santa Cruz Biotechnology and may not have been processed using an automated or optimized protocol [20]. In our study, by contrast, we used the JC8 clone developed by Agilent/Dako and performed staining on the Agilent/Dako Autostainer using a fully automated and standardized protocol. Optimization of the staining platform, in addition to the primary antibody itself, contributed to the improved performance of the JC8 clone in our setting. Thavaraj et al. conducted a comparative study using 170 formalin-fixed paraffin-embedded specimens of OPSCC to evaluate the performance of the Leica 6H12 (RTU) clone versus the Roche E6H4 (CINtec) clone [21]. They reported an overall concordance rate of 98.7–98.8%, with both positive and negative agreement rates ranging from 98.6% to 98.9%. Concordance with HPV molecular testing, including in situ hybridization (ISH) and polymerase chain reaction (PCR), was also high, ranging from approximately 86% to 94%. Based on these findings, the authors concluded that the Leica 6H12 clone demonstrated excellent concordance with E6H4 and could be considered a reliable and practical alternative [21]. These results are consistent with our findings in gynecologic tumors, further supporting the interchangeability of the 6H12 and E6H4 clones in p16 IHC applications. In recent years, additional p16 antibody clones beyond the three evaluated in our study have been developed. For instance, Angelico et al. reported the BC42 clone as a promising new candidate for use in gynecologic tumors [22]. In their study, the BC42 clone demonstrated a staining performance comparable to that of E6H4 in terms of positivity rates and specificity across 12 tumor types, including cervical, endometrial, and ovarian neoplasms. Given the relatively high cost of the E6H4 clone (CINtec), BC42 may be a practical alternative for p16 IHC, alongside JC8 and 6H12.

Initially, we compared the staining performance of three p16 clones using whole slide sections from 42 cases, predominantly cervical carcinomas. For further validation, we analyzed additional tissue microarrays comprising 44 endometrial carcinomas and 90 ovarian carcinomas, tumor types that were underrepresented in the initial cohort. The p16 staining patterns (absence, mosaic pattern, and block-type pattern) were completely concordant among the three clones, providing further evidence in support of the findings obtained from whole slide evaluation. These findings suggest that the three p16 clones can be used interchangeably not only as surrogate markers for high-risk HPV in cervical tumors, but also as useful markers for the diagnosis of high-grade carcinomas in the endometrium and ovary. Although TMAs are methodologically more vulnerable than whole slide sections to intratumoral heterogeneity of molecular expression, this limitation is unlikely to substantially affect the interpretation of our results given the specific aim of this study, which was to compare staining performance among the clones.

In our study, the interpretation of p16 IHC demonstrated excellent interobserver reproducibility. In general, the assessment of p16 IHC is relatively straightforward, and previous reports have suggested that its application improves diagnostic agreement in morphologically challenging lesions such as squamous intraepithelial lesions (SIL), where interobserver variability is often problematic. For example, in a study by Klaes et al. involving 194 cervical conization specimens, five pathologists independently evaluated hematoxylin and eosin (H&E)-stained slides and p16 IHC-stained slides [23]. The interobserver agreement improved markedly from a κ value of approximately 0.60 with H&E alone to 0.91 (95% CI: 0.84–0.99) with the addition of p16 IHC [23]. A similar trend was observed in the large-scale CERTAIN trial, which analyzed more than 11,000 cervical biopsies evaluated by approximately 70 pathologists across the United States [24]. In that study, the κ statistic improved from 0.58 (moderate agreement) with H&E alone to 0.73 (substantial agreement) with the addition of p16 IHC. These findings support the well-established utility of p16 IHC in the diagnosis of cervical intraepithelial neoplasia [24]. Comparable benefits have also been reported in the evaluation of anal squamous lesions [25].

More recently, Tao et al. evaluated nearly 200 cervical biopsy specimens using two experienced pathologists and reported a 92.5% concordance rate for p16 positivity/negativity, with a Cohen’s κ of 0.85 [26]. In contrast to other biomarkers such as HER2 and PD-L1, where interobserver reproducibility remains a recognized challenge [27,28], prior studies and our findings suggest that the interpretation of p16 positivity—specifically, block-type staining—is relatively straightforward and reproducible, regardless of the observer’s level of experience. This implies that the learning curve for accurate p16 IHC assessment may be less steep than for other immunohistochemical markers.

In our current study, a small number of non–block-type staining patterns were observed in cells other than those expected to show definitive block positivity or complete negativity. However, none of these findings interfered with the interpretation of p16 status in tumor cells. According to the NordiQC report, weak staining in scattererd fibroblasts, macrophages, vascular endothelial cells, and benign epithelial cells was anticipated and considered acceptable. Previous studies have consistently reported that the E6H4 clone exhibits the lowest levels of background and non-specific staining. For example, Shelton et al., in their comparative study of OPSCC specimens using E6H4, JC8, and G175-405, explicitly stated that E6H4 showed the least background and non-specific staining [20]. Similarly, the NordiQC—one of the leading external quality assurance programs for IHC in Europe—has reported a 100% pass rate for the Ventana RTU version of E6H4, indicating minimal concern regarding non-specific reactivity [16].

JC8 and its closely related clone JC2 have also performed well in NordiQC assessments, along with other clones such as MX007 and 6H12 [16]. In particular, JC2 has been reported to produce evident block-type positivity with minimal background staining in high-grade squamous intraepithelial lesions (HSIL) and squamous cell carcinomas. The 6H12 clone (Leica RTU) has similarly been endorsed by NordiQC as a recommended clone, with background staining remaining within acceptable limits [16]. Notably, some reports suggest that 6H12 outperforms E6H4 on certain platforms, such as the Leica BOND III, where E6H4 has demonstrated reduced performance [21].

In contrast, several reports have highlighted significant issues with non-specific staining using the G175-405 clone (BD) [15]. NordiQC has cited frequent background and false-positive staining with this clone, including artifactual staining of stromal and epithelial elements in tonsil control tissues. Studies involving OPSCC specimens have also noted lower κ values and weaker staining intensity with G175-405, raising concerns about its reliability due to excessive background staining [15].

p16 IHC is widely used in the field of gynecologic pathology, and numerous studies have examined its diagnostic and prognostic utility [1]. It is generally recognized that different antibody clones may exhibit varying staining characteristics, as has been observed with other biomarkers such as HER2 and PD-L1, where differences in antibody selection can significantly impact reported positivity rates and study interpretations. However, based on the findings of the present study, when staining is performed using standardized, automated platforms and manufacturer-recommended protocols, the results obtained with the E6H4, JC8, and 6H12 clones appear to be largely interchangeable.

Given that the E6H4 clone is relatively expensive, our results also support the potential use of more cost-effective alternatives, such as JC8 and 6H12, particularly when appropriately validated as laboratory-developed tests (LDTs) within individual institutions. Moreover, in the event of manufacturing issues, supply chain disruptions, or other unforeseen circumstances that limit the availability of a specific clone, our data may serve as a practical reference for laboratories seeking rapid and reliable substitution strategies as part of risk management planning.

However, certain limitations of our study must be acknowledged, and the findings should not be overgeneralized. Our analysis was limited to three relatively recent p16 antibody clones and a broader range of commercially available p16 IHC clones [1] was not included. Moreover, even when the exact clone name is used, differences in manufacturers, staining platforms, and protocol conditions may lead to variability in staining outcomes. In addition, it is recognized that IHC results are influenced not only by the choice of primary antibody clone but also by a variety of pre-analytical factors (e.g., formalin fixation conditions) and post-analytical variables (e.g., interpretation criteria) [19]. Therefore, when implementing p16 IHC in clinical laboratories, it is essential to perform appropriate in-house validation and to participate in external quality assurance programs, such as those offered by CAP and NordiQC, to ensure diagnostic reliability and consistency. Additional limitations include the small number of vulvar carcinoma cases, the absence of HPV status data, and the potential for selection bias due to the non-consecutive nature of the cohort. These factors should be taken into account when interpreting the results.

## 6. Conclusions

In conclusion, we compared the staining performance of three fully automated p16 IHC antibody clones—E6H4, JC8, and 6H12—across 176 gynecologic tumor specimens, including those from the cervix, endometrium, ovary, and vulva. Our study demonstrated excellent concordance and interobserver reproducibility among these clones. These findings may serve as a valuable reference for comparing results across studies utilizing different p16 IHC clones. They may aid clinical laboratories in selecting cost-effective and diagnostically reliable p16 IHC assays.

## Figures and Tables

**Table 1:** Summary of 42 cases assessed based on whole slide sections.

Characteristics	N (%)
Total	42 (100)
Organ	
Uterine cervix	30 (71)
Uterine corpus	4 (9.5)
Vulva	6 (14)
Ovary	2 (4.8)
Specimen type	
Surgical specimen	26 (62)
Biopsy specimen	16 (38)
p16 (E6H4) immunohistochemistry	
Positive	21 (50)
Negative	21 (50)
Diagnosis	
Squamous cell carcinoma, HPV-associated	12 (29)
Cervicitis	6 (14)
High-grade squamous intraepithelial neoplasia/CIN3	4 (9.5)
Squamous cell carcinoma, HPV-independent	4 (9.5)
Adenocarcinoma in situ, HPV-associated	2 (4.8)
Adenocarcinoma in situ, HPV-independent, gastric-type	2 (4.8)
Adenocarcinoma, HPV-associated	2 (4.8)
Adenocarcinoma, HPV-independent, gastric type	2 (4.8)
High-grade serous carcinoma	2 (4.8)
Neuroendocrine carcinoma	2 (4.8)
Serous carcinoma	2 (4.8)
Clear cell carcinoma	1 (2.4)
Endometrioid carcinoma	1 (2.4)

**Table 2:** Summary of 134 cases analyzed using tissue microarrays.

Characteristics	N (%)
**Endometrial cancer**	44 (100)
Endometrioid carcinoma, grade 1/2	17 (39)
Endometrioid carcinoma, grade 3	16 (36)
Serous carcinoma	5 (11)
Clear cell carcinoma	3 (6.8)
Carcinosarcoma	3 (6.8)
**Ovarian cancer**	90 (100)
High-grade serous carcinoma	35 (39)
Low-grade serous carcinoma	3 (3.3)
Clear cell carcinoma	27 (30)
Endometrioid carcinoma	20 (22)
Mucinous carcinoma	5 (5.6)

**Table 3:** Details of p16 immunohistochemistry using three different ready-to-use antibodies.

Characteristics	E6H4	JC8	6H12
Vendor	Ventana/Roche	Agilent/Dako	Leica
Product No.	705-4713	GA783	PA0016
Type of antibody	Mouse monoclonal	Mouse monoclonal	Mouse monoclonal
Target epitope	Human p16^INK4a^, amino acids 134-156	Human p16^INK4a^, recombinant full-length human p16	Human p16^INK4a^ epitope not fully disclosed
Isotype	IgG2a	IgG2a	IgG2b
Dilution	Prediluted	Prediluted	Prediluted
Antigen retrieval	HIER using CC1 64 min. at 95 °C	HIER using EnVision FLEX High pH 30 min. at 97 °C	HIER using BERS2 20 min. at 100 °C
Primary antibody incubation time	16 min	20 min	15 min
Autostainer	BenchMark Ultra	Dako Omnis	Leica BOND III
Detection system	UltraView	EnVision Flex+	Bond Polymer Refine Detection (DS9800)
Clinical Validation	FDA-approved as part of CINtec^®^ Histology kit	RUO	RUO

Abbreviations: HIER, heat-induced epitope retrieval; RUO research use only.

**Table 4:** Concordance of p16 IHC results between three clones in 42 cases analyzed using whole slide sections.

		JC8	
		Positive	Negative
E6H4	Positive	21	0
	Negative	0	21
		6H12	
		Positive	Negative
E6H4	Positive	21	0
	Negative	0	21
		6H12	
		Positive	Negative
JC8	Positive	21	0
	Negative	0	21

**Table 5:** Concordance of p16 IHC results between three clones in 134 cases analyzed using tissue microarrays.

		JC8		
		Positive Block staining	Negative Mosaic staining	Negative Absence
E6H4	Positive Block staining	40	0	0
	Negative Mosaic staining	0	68	0
	Negative Absence	0	0	26
		6H12		
		Positive		
E6H4	Positive Block staining	40	0	0
	Negative Mosaic staining	0	68	0
	Negative Absence	0	0	26
		6H12		
		Positive		
JC8	Positive Block staining	40	0	0
	Negative Mosaic staining	0	68	0
	Negative Absence	0	0	26

**Table 6:** Interobserver agreements of p16 immunohistochemistry between two pathologists.

		Pathologist B	
		Positive	Negative
Pathologist A	Positive	30	0
	Negative	0	30

## Data Availability

The data presented in this study are available on request from the corresponding author because data sharing was not covered by the patient consent obtained for this study.
